# Establishing key components of yoga interventions for reducing depression and anxiety, and improving well-being: a Delphi method study

**DOI:** 10.1186/s12906-015-0614-7

**Published:** 2015-03-26

**Authors:** Michael de Manincor, Alan Bensoussan, Caroline Smith, Paul Fahey, Suzanne Bourchier

**Affiliations:** National Institute of Complementary Medicine (NICM), University of Western Sydney, Campbelltown Campus, Locked Bag 1797, Penrith, NSW 2751 Australia

**Keywords:** Yoga, Mental health, Depression, Anxiety, Well-being, Consensus, Intervention protocol, Delphi method

## Abstract

**Background:**

Previous research suggests benefits of yoga in reducing depression and anxiety. However, common concerns in reviews of the research include lack of detail, rationale and consistency of approach of interventions used. Issues related to heterogeneity include amount, types and delivery of yoga interventions. This study aims to document consensus-based recommendations for consistency of yoga interventions for reducing depression and anxiety.

**Methods:**

The Delphi method was used to establish consensus from experienced yoga teachers. Thirty-three eligible teachers were invited to participate, from four different countries. Two rounds of an online survey were sent to participants. The first round sought initial views. The second round sought consensus on a summary of those views. Survey questions related to frequency and duration (dosage) of the yoga, approaches and techniques to be included or avoided, and training and experience for yoga teachers.

**Results:**

Twenty-four teachers agreed to participate. Eighteen completed the second round (*n* = 18). *General consensus* (>75% of participants in agreement) was achieved on parameters of practice (dosage): an average of 30 to 40 minutes, to be done 5 times per week, over a period of 6 weeks. Numerous recommendations for yoga techniques to include or avoid were collected in the first round. The second round produced a consensus statement on those recommendations. Breath regulation and postures were considered very important or essential for people with depression; and relaxation, breath regulation and meditation being very important or essential for people with anxiety. *O*ther recommended components also achieved consensus. There was also *general consensus* that it is very important or essential for teachers to have a minimum of 500 training hours over 2 years, at least 2 years teaching experience, training in developing personalised yoga practices, training in yoga for mental health, and professional supervision or mentoring.

**Conclusions:**

The Delphi process has achieved a consensus statement on the application of yoga for reducing anxiety and depression. This consensus provides a checklist for identification of commonalities and evaluation of past research. Future research can proceed to develop and evaluate consensus-based yoga intervention protocols for the reduction of anxiety and depression, and improvements in well-being.

**Electronic supplementary material:**

The online version of this article (doi:10.1186/s12906-015-0614-7) contains supplementary material, which is available to authorized users.

## Background

Mental health concerns affect many Australians. In 2011–12, 3 million Australians (13.6%) reported having a mental and/or behavioural condition. Mood (affective) disorders, including depression, were the most prevalent (2.1 million people or 9.7% of the population), followed by anxiety related problems (850,100 people or 3.8%) [1].

Current treatments for depression and anxiety include a range of pharmaceutical medications, psychological therapies, electro-convulsive therapy, complementary and lifestyle interventions, and a combination of these [2-10]. Questions about side effects, placebo effects, cost effectiveness, individual choice, access to services, compliance, ethics and long term benefits, remain unclear.

There is increasing interest in the potential role of yoga as an intervention for mental health concerns [5,7,11-14]. Yoga is a wholistic multi-dimensional system of health and well-being that focuses on the mind and its functions, with multi-component mind-body practices, including four main categories of practice: i) physical postures and movement; ii) breathing exercises; iii) relaxation; and iv) mindfulness and meditation. Other aspects of yoga practice include cultivation of positive values, thoughts and attitudes, and lifestyle factors. It is a multi-dimensional intervention that can be tailored to the needs of each individual.

Yoga practices may be done individually or in group classes. Yoga may be appealing to people who are concerned with the stigma associated with conventional mental health treatments, or with the narrow focus of a diagnosis-treatment approach that defines much of current mental illness treatment, and are instead attracted to a broader focus on mind-body or lifestyle interventions, for living a healthier, happier and flourishing life [15]. Yoga is also increasingly available, and may prove cost-effective compared to other types of mental health treatments. Yoga could also be used as an adjunct to conventional treatments. A yoga practice can be modified for people with specific concerns, such as pregnant women, who are frequently reluctant to use medications. When used to assist people in treatment or recovery from injury, illness or disability, it is often referred to as yoga therapy [16,17]. Finally, yoga may simultaneously have a range of other desirable effects in general health and well-being. Many yoga practitioners view yoga as a way to promote both physical and mental health, rather than a treatment for specific illness [12].

Prior research, including several systematic reviews, suggests benefits of yoga in reducing depression and anxiety [15,18-27]. However, the variety in severity of symptoms, types of interventions, and limitations of trial methodology and reporting, suggest that the findings must be interpreted with caution [28,12]. A common concern in the research reviews is the considerable heterogeneity and lack of detail, rationale and consistency of approach in the types of yoga-based interventions between the various studies [29,22,28,12]. As yoga includes a broad range of practice and techniques, variability and lack of details about the interventions included in the studies makes it very difficult to draw generalizable conclusions of the benefits of yoga and identification of effectiveness of key components of interventions.

The aim of this study is to document ‘expert opinion’ in use of yoga interventions for depression and anxiety, by drawing upon the cumulative knowledge and experience of experienced practitioners in the field. We aim to produce a consensus statement documenting recommended elements of yoga interventions for these conditions, which can later be used to guide and evaluate evidence-based research in the area.

## Methods

A modified Delphi technique was used [30]. The Delphi technique uses anonymity, iteration and controlled feedback to arrive at a consensus; it is economical and not constrained by geographical limitations. This method gives equal weighting to views of each participant reducing the risk of one particular participant or view dominating. The study was given ethics approval by the University of Western Sydney, Human Research Ethics Committee (approval number H9523).

### Delphi process and participant selection

Potential components for yoga-based interventions were drawn together from literature and experienced clinicians to generate a questionnaire. Thirty-three yoga teachers/therapists working in the field of yoga and mental health were identified by the primary researcher through literature reviews and professional networks, and invited by email to participate in the study. Eligibility for participation included the equivalent of minimum training required for Senior or Level 3 membership of Yoga Australia (1000 hours training and 10 years teaching experience; double the training requirements for E-RYT 500 with Yoga Alliance, USA); specialized training and experience in teaching people one-to-one and developing personalised yoga practices for individual needs; and a minimum of 5 years experience working as a yoga teacher/therapist with people experiencing mental health concerns including depression or anxiety. Twenty-four of the 33 agreed to participate. Two rounds of an online questionnaire using Survey Monkey were sent to participants over a period of 5 months, from May to September, 2012. Survey questions related to expected benefits of yoga for various degrees of symptom severity, recommended frequency and duration of yoga practice (“dosage” of yoga) required to achieve expected benefit, approaches and techniques to be included or avoided, and required training and experience for yoga teachers. Questionnaires used are provided as Additional files 1 and 2. The first round sought initial views of participants, which were summarised and redistributed to the group. The second round sought responses to the relative importance of each item, which were rated as:

### Not recommended not important somewhat important very important essential

Responses were collated as frequency per response category. Level of consensus is measured as the number ratings given in each response category. We define *general consensus* as more than 75% (14/18) of participants rating in a particular response category. Results are reported as a summary of consensus from both rounds of questionnaires.

## Results

Thirty-three eligible yoga teachers, experienced in the field of mental health, were invited to participate in round one of the survey. Twenty-four (19 female and 5 male) agreed to participate, and eighteen (13 female and 5 male) completed the second round (*n* = 18). Participants were from four different countries - Australia (14), India (3), Switzerland (1), and USA (6). Ten participants were also mental health professionals (psychologist or medical doctor), although this was not an eligibility requirement.

### Practice parameters (or “dosage” of the yoga intervention)

There was *general consensus* that doing a suitable individually tailored yoga practice under the guidance of a teacher required a minimum of 15 minutes per session to be of any benefit, and that an average of 30 to 40 minutes was recommended to maximize benefit. Also, the *general consensus* was for a minimum of 4 sessions per week to achieve benefit, and preferably an average of 5 to 6 times per week to achieve maximum benefit, and to be done over a minimum period of 6 weeks (Table 1).

**Table 1:** ***General consensus*** **achieved on recommended parameters (“dosage”) of yoga practice**

**Practice parameter**	**Recommended minimum**	**Recommended average**
**Duration of each session**	**15 minutes**	**30 to 40 minutes**
**Frequency of sessions**	**4 sessions per week**	**5 to 6 sessions per week**
**Duration over time**	**6 weeks**	**n/a**

### Practice recommendations - Yoga protocols

#### Main components of recommended yoga practice and their relative importance

There was *general consensus* that any or all of the four main components of yoga practice (postures, regulation of breathing, relaxation, and meditation) would be recommended in an individually tailored yoga practice for reducing depression or anxiety. There was also *general consensus* that breath regulation was very important or essential in yoga practices for reducing both depression and anxiety, and that yoga postures were very important or essential for reducing depression, and somewhat or very important, but less essential, for reducing anxiety. And, there was *general consensus* that relaxation and meditation were very important or essential for reducing anxiety, and a mixture of somewhat important, very important or essential, without *general consensus* on their relative importance for reducing depression (Tables 2 and 3).

**Table 2:** **Relative importance of components of yoga practice for reducing depression or anxiety**

	***General consensus*** **on being very important or essential**	
**Component of yoga practice**	**for reducing depression**	**for reducing anxiety**
Breath regulation	Yes	Yes
Postures	Yes	No*
Relaxation	No	Yes
Meditation	No	Yes

*Note: there was *general consensus* that postures were a somewhat or very important, but less essential component for reducing anxiety.

**Table 3:** **Consensus on importance of approaches and techniques for reducing depression or anxiety, and improving positive emotions and well-being**

**Round 1 - recommendations**	***Round 2 -***	
	***General consensus on importance***	
**Breath regulation - approaches & techniques**	***For reducing***	***For reducing***
	***Depression***	***Anxiety***
Abdominal breathing	Very important/essential	Very important/essential
Focus on inhalation	Important/less essential	No recommendation
Focus on exhalation	No recommendation	Very important/essential
Comfortable holding after inhalation	Important/less essential	Important/Essential to AVOID
Comfortable holding after exhalation	No recommendation	Important/less essential
Alternate nostril breathing	No recommendation	Important/less essential
Right nostril breathing, especially on inhalation	Important/less essential	No recommendation
Left nostril breathing, especially on exhalation	No recommendation	Important/less essential
Cooling breath (*sitali*)	Less or not important	Important/less essential
Rapid breathing techniques, such as *kapalabhati*	Less or not important	Important/Essential to AVOID
“humming bee” breath (*bhramari*)	No recommendation	Very important/essential
Regulating the breath to be calm and steady	No recommendation	Very important/essential
**Postures - approaches & techniques**	***For reducing***	***For reducing***
	***Depression***	***Anxiety***
Coordinated flow of breath with movement	Very important/essential	Very important/essential
Chest and heart opening, backward bending postures and movements, that also focus on inhalation	Important/less essential	No recommendation
Moving repetition of postures (rather than long holding)	Important/less essential	No recommendation
Dynamic sequences of postures, including sun salutations	Important/less essential	No recommendation
A range of different postures, to keep it interesting	Important/less essential	No recommendation
Postures that have a calming effect, rather than energising	No recommendation	Very important/essential
Resting, relaxing or restorative postures	NO CONSENSUS	Important/less essential
Forward-bending postures	No recommendation	Important/less essential
Postures and movements that focus on exhalation	No recommendation	NO CONSENSUS
Simple, gentle sequences of postures	No recommendation	NO CONSENSUS
**Relaxation - approaches & techniques**	***For reducing***	***For reducing***
	***Depression***	***Anxiety***
Focus on abdominal breathing, lengthening exhale	Not considered	Very important/essential
Active focus on physical body (e.g. body-scan; progressive muscle relaxation), to shift focus away from mind and thoughts	Very important/essential	NO CONSENSUS
Done with visualisations, that are positive, expansive and energising, e.g. sun, open space.	Very important/essential	No recommendation
Done with visualisations, that have a calming effect	No recommendation	NO CONSENSUS
Using guided relaxation techniques, rather than self directed	Important/less essential	No recommendation
Restorative (passive-supported) postures	NO CONSENSUS	Important/less essential
With legs elevated	NO CONSENSUS	NO CONSENSUS
Resting between and after postures	NO CONSENSUS	NO CONSENSUS
**Meditation - approaches & techniques**	***For reducing***	***For reducing***
	***Depression***	***Anxiety***
Mindfulness (learning to focus attention on observing the present experience, without judgement)	Very important/essential	Very important/essential
Something for the mind to do and focus on, rather than just observation (e.g. counting, repeated words or phrases (mantra); visualisation; image or symbol; candle gazing; smiling heart)	Very important/essential	Important/less essential
Active meditations, e.g. moving, chanting, guided visualisations	Very important/essential	Important/less essential
A concept, idea or value, such as something positive, energising, confidence building, gratitude.	Very important/essential	No recommendation
A concept, idea or value, such as something positive, calming, confidence building, gratitude.	No recommendation	Important/less essential
**Other components of yoga practice**	***For reducing***	***For reducing***
	***Depression***	***Anxiety***
Cultivation of positive values, attitudes and behaviours (including gratitude, kindness, compassion, forgiveness)	Very important/essential	Very important/essential
Awareness of negative sensory input (including TV, movies, music, literature, multi-media, news)	Very important/essential	Very important/essential
Formulation of meaningful affirmations and intentions (samkalpa)	Important/less essential	Important/less essential
Visualisation and symbolic imagery techniques (bhavana)	Important/less essential	Important/less essential
Sound or chanting (from any suitable language or culture)	Important/less essential	Important/less essential
Spirituality and prayer	Important/less essential	Important/less essential
Repetition of meaningful words or phrases (mantra)	Important/less essential	Important/less essential
Symbolic gesture (nyasa & mudra)	Important/less essential	Important/less essential
**Other components of yogic lifestyle**	***For reducing***	***For reducing***
	***Depression***	***Anxiety***
Positive relationships	Very important/essential	Very important/essential
Developing self-empowering knowledge	Very important/essential	Very important/essential
Social involvement and support - linking with a supportive community (sanga)	Very important/essential	Very important/essential
Lifestyle factors, including diet, smoking, drugs and alcohol, sleep, work, exercise	Very important/essential	Very important/essential
Exposure to sunlight and natural environments	Very important/essential	Very important/essential
Service to others, including volunteer work	Important/less essential	Important/less essential
Pleasing environment, free from clutter, with good ventilation and natural light	Important/less essential	Important/less essential
Education about yoga teachings, and the potential benefits of yoga	Important/less essential	Important/less essential
Group yoga classes	Important/less essential	No recommendation

#### Recommended approaches and techniques

Numerous and detailed recommendations for particular approaches and techniques to include or avoid for people with depression or anxiety were given in the round 1 questionnaire. These were summarised and their relative importance rated in round 2. Some approaches and techniques were recommended for reducing both depression and anxiety. Others were recommended for reducing either depression or anxiety.

There was *general consensus* that yoga is most beneficial when the different components or techniques of yoga practice are used with an *integrated and individualised approach* (i.e. in combination or conjunction with each other, according to the suitability for each individual).

There was also *general consensus* that recommended approaches and techniques for reducing depression or anxiety were the same as recommendations for increasing positive emotions and well-being.

### Recommended approaches and techniques for reducing BOTH depression and anxiety

There was *general consensus* that the following approaches and techniques would be recommended for reducing both depression and anxiety.

- *Regulation of breathing*: Relaxed abdominal-diaphragmatic breathing is very important or essential.
- *Postures:* Coordinated flow of breath with movements in postures is very important or essential.
- *Relaxation:* There was no particular approach to relaxation techniques that was considered very important or essential for reducing both depression and anxiety.
- *Meditation*: Even though meditation in general was considered less essential for reducing depression, mindfulness techniques (learning to focus attention on observing the present experience, without judgement) was considered very important or essential for reducing both depression and anxiety. Active meditation techniques that give the mind something to do or focus on (rather than just observation; e.g. counting, repeated words or phrases (*mantra*); visualisation; image or symbol; candle gazing; or smiling heart), or other active meditation techniques (such as moving, chanting or guided meditations), were also considered very important or essential for reducing depression, and somewhat or very important, but less essential for reducing anxiety.

*General consensus* was also achieved on recommendations for other components of yoga practice and yogic lifestyle for reducing both depression and anxiety.

e. *Other components of yoga practice:* Cultivation of positive values, attitudes and behaviours, including gratitude, kindness and compassion; and awareness of negative sensory input (*pratyahara*), including TV, movies, music, multi-media, or news, were both considered very important or essential. Other components were considered somewhat or very important, but less essential, including formulation of meaningful affirmations and intentions (*samkalpa*); visualisation and symbolic imagery techniques (*bhavana*); use of sound or chanting (from any suitable language or culture); cultivation of spirituality or prayer (*isvarapranidhana*); repetition of meaningful words or phrases (*mantra*); and meaningful symbolic gestures (*nyasa & mudra*).
f. *Other components of yogic lifestyle:* Cultivating positive relationships; education and developing self-empowering knowledge; social involvement and support (linking with a supportive community - *sanga*); lifestyle factors including diet, smoking, drugs, alcohol, sleep, work and exercise; and exposure to sunlight and natural environments, were each considered very important or essential. Service to others (including volunteer work); pleasing environment, free from clutter, with good ventilation and natural light; and education about yoga teachings and potential benefits of yoga, were also considered somewhat or very important, but less essential.

### Additional approaches and techniques recommended for reducing depression.

Along with recommendations for reducing both depression and anxiety, there was *general consensus* on recommendation of the following approaches and techniques for reducing depression.

- *Regulation of breathing*: A focus on inhalation, comfortable holding after inhalation, and right nostril breathing, especially on inhalation, were considered somewhat or very important, but less essential. Cooling breath (*sitali*) and rapid breathing techniques (such as *kapalabhati*) were considered as less or not important.
- *Postures:* Moving repetition of postures, rather than longer holding; chest and heart opening, backward bending postures and movements, that also focus on inhalation; dynamic sequences of postures, including sun salutations; doing a range of different postures to keep it interesting; were each considered somewhat or very important, but less essential.
- *Relaxation*: Active focus on the physical body (e.g. body awareness scan, progressive muscle relaxation) to shift focus of attention away from mind and thoughts; visualisations that are positive and energising (e.g. sun, open space) were considered very important or essential; and guided relaxation techniques, rather than self-directed, were somewhat or very important, but less essential.
- *Meditation*: Giving a specific concept, idea or value to focus on, that is positive, energising, or confidence building for the person was considered very important or essential.
- *Other components of yoga practice:* There were no additional components of yoga practice recommended for reducing depression.
- *Other recommended yogic-lifestyle factors*: Attending group yoga classes was considered somewhat or very important, but not essential.

### Additional approaches and techniques recommended for reducing anxiety

Unlike depression, the main components of yoga practice that were considered most important for reducing anxiety were relaxation, breath regulation, and meditation. Postures were considered important, but less essential.

Along with recommendations for reducing both depression and anxiety, there was *general consensus* on the following approaches and techniques for reducing anxiety (Table 4).

**Table 4:** **Consensus on approaches and techniques to AVOID, for people with depression or anxiety**

**Round 1 - recommendations to avoid**	***Round 2 - General consensus achieved:***	
	***For people with Depression***	***For people with Anxiety***
Meditation practices without any specific focus (such as emptiness or inner silence meditation)	Important or Essential to AVOID	Important or Essential to AVOID
Rapid breathing techniques, such as *kapalabhati* (if anxiety or history of trauma is also present)	Important or Essential to AVOID	Important or Essential to AVOID
Strong, vigorous or strenuous postures	Important or Essential to AVOID	Important or Essential to AVOID
Yoga done in heated, crowded, enclosed spaces	No consensus	Important or Essential to AVOID
Any practices which are too introspective	No consensus	Important or Essential to AVOID
Techniques that emphasise ability, accomplishment, performance, competition	No recommendation	Important or Essential to AVOID
Techniques that require difficult and complex instructions	No recommendation	Important or Essential to AVOID
Comfortable holding after inhalation	No recommendation	Important or Essential to AVOID

- *Relaxation*: A focus on relaxed abdominal breathing, and lengthening the exhale in relaxation techniques are considered very important or essential. Restorative (passive-supported) postures are considered somewhat or very important, but less essential. And, other components with a mixture of somewhat important, very important, or essential, without *general consensus* on how important, included an active focus on the physical body during relaxation (e.g. body awareness scan, progressive muscle relaxation) to shift focus of attention away from mind and thoughts; and visualizations that have a calming effect for the person.
- *Regulation of breathing*: Lengthening the exhalation, calm and steady breathing, and “humming bee” breathing, were considered very important or essential. And, cooling breath (*sitali)*, alternate nostril breathing (*nadi shodhana*), left nostril breathing, especially on exhalation, and comfortable holding after exhalation were each considered somewhat or very important, but less essential.
- *Meditation*: Giving a specific concept, idea or value to focus on, that is positive, calming, or confidence building for the person was considered somewhat or very important, but less essential.
- *Postures*: Doing yoga postures that have a calming effect, rather than energising, was considered very important or essential. And, forward bending postures, and resting, relaxing or restorative postures, were considered somewhat or very important, but less essential. Other approaches were recommended with a mixture of somewhat important, very important, or essential, but without *general consensus* on how important, included, simple gentle sequences of postures, and postures that focus on exhalation.
- *Other components of yoga practice and yogic-lifestyle:* There were no additional components of yoga practice or yogic-lifestyle recommended for reducing anxiety.

#### Approaches and techniques recommended to AVOID

There was *general consensus* that it is important, very important or essential to avoid a number of yoga practices or techniques for both depression and anxiety, and some techniques particularly for anxiety.

- *Recommended to AVOID for BOTH depression and anxiety:* Rapid breathing techniques should be avoided for anxiety, and for depression if a history of anxiety or trauma is also indicated. It is also recommended to avoid meditation practices without a specific focus (such as emptiness or inner silence) for both depression and anxiety; and strong or strenuous postures, if there is low motivation or energy.
- *Additional techniques to AVOID for anxiety:* yoga done in heated, crowded or enclosed spaces, and breath regulation that focuses on holding after inhalation should be avoided for people with anxiety. And, techniques and practices that emphasize ability, accomplishment or competition, or require difficult and complex instructions, should also be avoided.

#### Training and experience of yoga teachers - Delivery of the yoga intervention protocols

*General consensus* was achieved in recommendations for training and experience of yoga teachers delivering the protocol for people with depression or anxiety. It was considered very important or essential for teachers to have a minimum of 500 training hours over 2 years, 2 years teaching experience, training in developing personalised yoga practices, training in yoga for mental health, and professional supervision or mentoring.

### Expected benefits

There was *general consensus* that a benefit of 50% to 90% (median 80%) reduction of symptoms of depression or anxiety, and associated increases in positive emotions and well-being, would be expected for a person with mild or moderate depression or anxiety doing a suitable, individually tailored yoga practice, based on these protocols, under the guidance of a suitably trained and experienced teacher, and generally done in conjunction with other forms of treatment or intervention. There was also *general consensus* that less benefit would be expected for more severe conditions.

## Discussion

The aim of this Delphi study was to identify by *general consensus* components of yoga practice that could be used in the development of intervention protocols and future research evaluating yoga for reducing depression or anxiety and improving well-being. The Delphi technique has successfully been used to reach consensus in medical and health services research [31], and complementary medicine treatment protocols, including yoga for musculoskeletal conditions [32], and acupuncture [33-35].

Yoga teachers from four different countries, experienced in the field of mental health participated in two rounds of an online survey. Moderate recruitment and completion rates indicate professional interest in the importance of addressing identified issues of heterogeneity in yoga interventions in prior research.

Survey questions related to three main areas of yoga intervention protocols: parameters of frequency and duration of yoga practice (or “dosage” of yoga); approaches and techniques to be included or avoided; and required training and experience for yoga teachers delivering the interventions. A fourth area was related to expected benefits from the interventions. *General consensus* (>75% agreement) was achieved on a number of items in each of these areas.

Interventions in prior research are predominantly comprised of group yoga classes, with varying frequency and duration, as well as style or type of yoga taught, and most yoga teachers are primarily trained to teach group yoga classes. In this study, there was *general consensus* that a suitable individually-tailored yoga practice, with lessons and guidance on a one-to-one basis from a suitably trained and experienced teacher, would be beneficial for people with depression or anxiety. This is consistent with current treatment recommendations on the importance of individually tailored treatment approaches in mental health [9]. People with depression may also gain benefit from group classes, perhaps due to the importance of social engagement. Individually-tailored or personal yoga practice is also consistent with historical teachings and practice of yoga [36], and the approach is sometimes known as ‘*viniyoga’* [36-38]*.* When yoga is used to assist people in treatment or recovery from injury, illness or disability, it is often referred to as ‘yoga therapy’ [17]. Whilst group classes are popular in many parts of the world today, they are a modern, western phenomenon. Personal or home-practice, has also emerged as a controversial and polarizing issue in another Delphi study on yoga interventions for musculoskeletal conditions [32]. This may be due to concerns about teaching supervision, compliance and monitoring of yoga practices done at home. When designing and describing yoga interventions, it is important to identify whether the yoga intervention is to be done in modern group classes, or as a personal practice, done at home. Further research to consider the relative effectiveness of each approach for reducing depression and anxiety is recommended.

A minimum amount (dosage) of 15 minutes (or preferably 30 to 40 minutes) per day, for a minimum of 4 (or preferably 5 to 6) times per week, over a minimum period of 6 weeks, is recommended as necessary to gain benefits. Whilst this seems to be a reasonable set of recommendations, adherence to this amount of regular yoga practice amongst people who experience symptoms of depression or anxiety, may be difficult and needs to be assessed for its practicality. Further research is also required to evaluate minimum, maximum and optimum amounts of yoga required to gain benefit, as well as differences amongst people with varying severity of conditions.

Again consistent with historical approaches to yoga, results from this study provide consensus for the development of a set of intervention protocol guidelines, to be used in a non-prescriptive *integrated and individualised approach* (i.e. in multi-dimensional combinations, according to the suitability for each individual). This may seem at odds with common approaches to best practice, practitioner training and delivery, and research requirements for standardised interventions that are replicable. However, this indicates the importance of a pragmatic balance between evidence-based standardization and individual participant needs, and quality multi-dimensional research methodology that explores optimum patient outcomes.

The strengths of this study include moderate recruitment and completion rates, the considerable expertise and experience of participants, the open scope for recommended intervention approaches, and the reproducible Delphi method. Limitations of the study are also noted. Firstly, whilst efforts were made to invite a reasonable number and representative sample of professionals in the field, the views of those who were invited and agreed to participate may not be representative of views generally held. We have little information about those who were invited but did not participate. Panelists were largely from Australia and USA, with little representation from European or other countries, including India. There may be different cultural and professional views from different parts of the world. Consideration must be given to the popular view that yoga in countries like USA and Australia, seems to emphasise the physical aspects of yoga postures, whereas in India and some European countries, there may be a more wholistic, philosophical or psychological approach to yoga. In contrast, consensus views from the current study did not emphasise the importance of yoga postures, and include a range of wholistic approaches and techniques. The sample size of this study was too small to support and scientifically valid comparisons. Regardless, the efficacy of the consensus-based intervention protocol from the current study requires evaluation in clinical trials. Also, whilst participants were identified and invited based on eligibility criteria, specific demographic details of participants, such as age, education level, and style(s) of yoga taught, were not included. Furthermore, whilst open format of the Delphi methodology is a strength for gathering views, numerous and varied responses for techniques to include in interventions were recommended, and some of these may have overlap and inconsistencies. The static process of survey methodology makes it difficult for participants to respond to each recommendation from other participants, which may effect consensus outcomes. Finally, *general consensus* that an average of 80% reduction of symptoms for mild or moderate severity of conditions is a substantial claim, and may require further clarification. In recommending key components and ‘dosage’ of yoga interventions, further consideration may need to be given to the severity and chronicity of conditions and how these are defined, particular symptoms being addressed, interactions with other forms of treatment including medications, and referral and source of diagnosis (self or health practitioner). In particular, assessment of severity of condition and distinctions between mild, moderate and severe conditions is somewhat subjective, and relies primarily on client self-reporting. In clinical practice, diagnosis by health practitioners often includes self-report measures such as the Depression, Anxiety and Stress Scale (DASS), where severity is based on conventional labels (normal, mild moderate, severe) and differences are essentially differences of degree, rather than allocation to discrete diagnostic categories postulated in classificatory systems such as the DSM [39]. This study did not include clarification of participants‘ conceptualisation or understanding of these factors, nor particular descriptions of symptom reductions. Further clarification of conditions and symptoms being assessed for expected benefits is required.

## Conclusion

The Delphi process has achieved a consensus opinion on the application of yoga-based interventions for reducing anxiety and depression, and improving the well-being of people with these conditions. This provides a checklist for the identification of key components, commonalities and differences in interventions in prior research in the field. Future research can proceed to develop and evaluate consensus-based yoga intervention protocols for the reduction of anxiety and depression, and improvements in well-being.

## Additional files

Additional file 1:
**Yoga for Mental Health_Delphi Questionnaire_Round1.**
Additional file 2:
**Yoga for Mental Health_Delphi Questionnaire_Round2.**
