# Supplementary material for: Establishing key components of yoga interventions for reducing depression and anxiety, and improving well-being: a Delphi method study
Source: BMC Complement Altern Med. 2015 Mar 26;15:85. doi: 10.1186/s12906-015-0614-7 (PMC4384362; doi:10.1186/s12906-015-0614-7)
Supplement: Additional file 1: — Yoga for Mental Health_Delphi Questionnaire_Round1. [file 12906_2015_614_MOESM1_ESM.pdf]

# Guidelines for Optimising Yoga Interventions in Mental Health

## Yoga for Reducing Depression and Anxiety, and Improving Well-Being

This study seeks to produce a consensus statement on best practice of yoga for people with depression or anxiety, based on the accumulated expertise of yoga teachers. Your participation and contributions are greatly appreciated.

By completing this questionnaire you will be participating in the development of guidelines to assist suitably trained yoga teachers to design individualised yoga practices for people with depression or anxiety. Such practices would be designed to alleviate depression and anxiety by increasing positive emotions and well-being.

Whilst we acknowledge that yoga practices are traditionally tailored to suit individual needs, there may be common factors related to suitable yoga practices for the needs of different people.

Completion of the questionnaire is taken as acknowledgement of your consent to participate.

Thank you again for your participation.

## Yoga for people with depression

Questions 1 to 3 relate to EXPECTED BENEFITS of yoga for people with depression

### 1. What degree of severity of depression do you think yoga would be beneficial for? (Please tick any or all that apply)

☐ Mild Depression ☐ Moderate Depression ☐ Severe Depression

Comments (optional)

### 2. How much reduction in depression would you expect to achieve from yoga?

| | Minor Reduction | Moderate Reduction | Considerable Reduction | Complete Reduction |
|---|---|---|---|---|
| Reduction of depression | ○ | ○ | ○ | ○ |

Comments (optional)

### 3. How much improvement in positive emotions and well-being would you expect a person with depression could achieve through yoga?

| | Minor Increase | Moderate Increase | Considerable Increase | Complete Increase |
|---|---|---|---|---|
| Increase in positive emotions & well-being | ○ | ○ | ○ | ○ |

Comments (optional)

## Yoga for people with anxiety

Questions 4 to 6 relate to EXPECTED BENEFITS of Yoga for people with anxiety

### 4. What degree of severity of anxiety do you think yoga would be beneficial for? (Please tick any or all that apply)

☐ Mild ☐ Moderate ☐ Severe

Comments (optional)

### 5. How much reduction of anxiety would you expect to achieve through yoga?

| | Mild Reduction | Moderate Reduction | Severe Reduction | Complete Reduction |
|---|---|---|---|---|
| Reduction of anxiety | ○ | ○ | ○ | ○ |

Comments (optional)

### 6. How much improvement in positive emotions and well-being would you expect a person with anxiety could achieve through yoga?

| | Mild Increase | Moderate Increase | Severe Increase | Complete Increase |
|---|---|---|---|---|
| Increase in positive emotions & well-being | ○ | ○ | ○ | ○ |

Comments (optional)

## Yoga for reducing Depression and Anxiety, and improving Well-Being

Questions 7 to 9 relate to parameters for the FREQUENCY AND DURATION of suitable yoga practices.

**7. How often would you recommend that a person with depression or anxiety does a personal yoga practice?**

AVERAGE number of sessions per week

MINIMUM number of sessions per week

MAXIMUM number of sessions per week

**8. What session length would you suggest is required for a regular personal yoga practice to have reasonable benefit in reducing depression or anxiety?**

AVERAGE number of minutes for practice

MINIMUM number of minutes for practice

MAXIMUM number of minutes for practice

**9. How many weeks of a personal yoga practice would you suggest is necessary for it to have some noticeable benefit in reducing depression or anxiety?**

MINIMUM number of weeks required

## YOGA FOR REDUCING DEPRESSION

Questions 10 to 14 relate to common factors in individual yoga practices for people with depression.

**10. Please describe any KEY FACTORS, approaches or techniques of the following, that you would recommend for people with DEPRESSION. Please use English, or explain Sanskrit terms.**

**You will also be asked to rank the relative importance of each of these, in the following question (Q11).**

**Key factors, approaches or techniques for reducing depression:**

Postures (asana)

Breath Regulation (pranayama)

Relaxation

Meditation

Other 1 (please specify)

Other 2 (please specify)

Other 3 (please specify)

Other 4 (please specify)

**11. Please rate the relative importance of each of the components you have described above (Q10), in a personal yoga practice for people with depression.**

| | Not Recommended | Not Important | Somewhat Important | Very Important | Essential |
|---|---|---|---|---|---|
| Postures (asana) | ○ | ○ | ○ | ○ | ○ |
| Breath Regulation (pranayama) | ○ | ○ | ○ | ○ | ○ |
| Relaxation | ○ | ○ | ○ | ○ | ○ |
| Meditation | ○ | ○ | ○ | ○ | ○ |
| Other 1 (as in Q10) | ○ | ○ | ○ | ○ | ○ |
| Other 2 (as in Q10) | ○ | ○ | ○ | ○ | ○ |
| Other 3 (as in Q10) | ○ | ○ | ○ | ○ | ○ |
| Other 4 (as in Q10) | ○ | ○ | ○ | ○ | ○ |

Comments (optional)

**12. What percentage of time would you recommend allocating for each component in a yoga practice for reducing depression? (Together, all responses should total 100%)**

Postures (asana)

Breath Regulation (pranayama)

Relaxation

Meditation

Other 1 (as in Q10)

Other 2 (as in Q10)

Other 3 (as in Q10)

Other 4 (as in Q10)

**13. Please describe any components or approaches of yoga that are important to AVOID for people with depression? Please explain your reasons.**

**14. Please describe any other factors that may be related to a yoga practice for reducing depression (e.g. diet, lifestyle, exercise, social involvement, etc)**

## YOGA FOR REDUCING ANXIETY

Questions 15 to 19 relate to common factors in individual yoga practices for people with anxiety.

**15. Please describe any KEY FACTORS, approaches or techniques of the following, that you would recommend for people with ANXIETY. Please use English, or explain Sanskrit terms.**

**You will also be asked to rank the relative importance of each of these, in the following question (Q16).**

**Key factors, approaches or techniques for reducing anxiety:**

Postures (asana)

Breath Regulation (pranayama)

Relaxation

Meditation

Other 1 (please specify)

Other 2 (please specify)

Other 3 (please specify)

Other 4 (please specify)

**16. Please rate the relative importance of each of these components in a personal yoga practice for people with anxiety.**

| | Not Recommended | Not Important | Somewhat Important | Very Important | Essential |
|---|---|---|---|---|---|
| Postures (asana) | ○ | ○ | ○ | ○ | ○ |
| Breath Regulation (pranayama) | ○ | ○ | ○ | ○ | ○ |
| Relaxation | ○ | ○ | ○ | ○ | ○ |
| Meditation | ○ | ○ | ○ | ○ | ○ |
| Other 1 (as in Q15) | ○ | ○ | ○ | ○ | ○ |
| Other 2 (as in Q15) | ○ | ○ | ○ | ○ | ○ |
| Other 3 (as in Q15) | ○ | ○ | ○ | ○ | ○ |
| Other 4 (as in Q15) | ○ | ○ | ○ | ○ | ○ |

Comments (optional)

**17. What percentage of time would you recommend allocating for each component in a yoga practice for reducing anxiety? (Together, all responses should total 100%)**

| | |
|---|---|
| Postures (asana) | |
| Breath Regulation (pranayama) | |
| Relaxation | |
| Meditation | |
| Other 1 (as in Q15) | |
| Other 2 (as in Q15) | |
| Other 3 (as in Q15) | |
| Other 4 (as in Q15) | |

**18. Please describe any components or approaches of yoga that are important to AVOID for people with anxiety? Please explain your reasons.**

**19. Please describe any other factors that may be related to yoga practice for reducing anxiety (e.g. diet, lifestyle, exercise, social involvement, etc)**

## YOGA FOR INCREASING POSITIVE EMOTION AND WELL-BEING

As well as reducing depression and anxiety, yoga may be viewed as a means to increasing positive emotion and well-being.

Questions 20 to 23 relate to common factors in a personal yoga practice for increasing positive emotions and well-being.

**20. Please describe any KEY FACTORS, approaches or techniques for the following, that you would recommend for people to increase positive emotions and well-being. Please use English, or explain Sanskrit terms.**

**You will also be asked to rank the relative importance of each of these, in the following question (Q21).**

**Key factors, approaches or techniques for increasing positive emotions and well-being:**

| | |
|---|---|
| Postures (asana) | |
| Breath Regulation (pranayama) | |
| Relaxation | |
| Meditation | |
| Other 1 (please specify) | |
| Other 2 (please specify) | |
| Other 3 (please specify) | |
| Other 4 (please specify) | |

**21. Please rate the relative importance of each of these components in a personal yoga practice for improving well-being.**

| | Not Recommended | Not Important | Somewhat Important | Very Important | Essential |
|---|---|---|---|---|---|
| Postures (asana) | ○ | ○ | ○ | ○ | ○ |
| Breath Regulation (pranayama) | ○ | ○ | ○ | ○ | ○ |
| Relaxation | ○ | ○ | ○ | ○ | ○ |
| Meditation | ○ | ○ | ○ | ○ | ○ |
| Other 1 (as in Q20) | ○ | ○ | ○ | ○ | ○ |
| Other 2 (as in Q20) | ○ | ○ | ○ | ○ | ○ |
| Other 3 (as in Q20) | ○ | ○ | ○ | ○ | ○ |
| Other 4 (as in Q20) | ○ | ○ | ○ | ○ | ○ |

Comments (optional)

**22. What percentage of time would you recommend allocating for each component in a yoga practice for increasing well-being? (Together, all responses should total 100%)**

Postures (asana)

Breath Regulation (pranayama)

Relaxation

Meditation

Other 1 (as in Q20)

Other 2 (as in Q20)

Other 3 (as in Q20)

Other 4 (as in Q20)

**23. Please describe any other factors that may be related to yoga practice for increasing positive emotion and well-being (e.g. diet, lifestyle, exercise, social involvement, etc)**

## TRAINING, EXPERIENCE AND MENTORING

Questions 24 to 27 relate to the training, experience and mentoring of the yoga teacher.

**24. When teaching yoga to people with mental health concerns, such as depression or anxiety, what do you recommend for MINIMUM training requirements?**

Minimum HOURS of training courses

Minimum YEARS of training courses

Minimum teaching EXPERIENCE (months or years?)

**25. In the context of teaching yoga to people with depression or anxiety, how important is SPECIFIC TEACHER TRAINING in developing INDIVIDUALISED YOGA PRACTICES, rather than general training for teaching group classes?**

| | Not Important | Somewhat Important | Very Important | Essential |
|---|---|---|---|---|
| Specific training for individualised yoga | ○ | ○ | ○ | ○ |

Comment (optional)

**26. How important is SPECIFIC TEACHER TRAINING in YOGA FOR MENTAL HEALTH?**

| | Not Important | Somewhat Important | Very Important | Essential |
|---|---|---|---|---|
| Specific training in yoga for mental health | ○ | ○ | ○ | ○ |

Comment (optional)

**27. How important is professional supervision or mentoring for yoga teachers who are working in the area of mental health?**

| | Not Important | Somewhat Important | Very Important | Essential |
|---|---|---|---|---|
| Supervision or Mentoring | ○ | ○ | ○ | ○ |

Comment (optional)

## Yoga for Mental Health and Well-Being

### 28. Any further comments?

Thank you for your participation. Again, your contribution is greatly appreciated.
We will contact you again soon for the next stage of the study.
