# Supplementary material for: Establishing key components of yoga interventions for reducing depression and anxiety, and improving well-being: a Delphi method study
Source: BMC Complement Altern Med. 2015 Mar 26;15:85. doi: 10.1186/s12906-015-0614-7 (PMC4384362; doi:10.1186/s12906-015-0614-7)
Supplement: Additional file 2: — Yoga for Mental Health_Delphi Questionnaire_Round2. [file 12906_2015_614_MOESM2_ESM.pdf]

Guidelines for Optimising Yoga Interventions in Mental Health - Second

Optimising Yoga for Reducing Depression & Anxiety, & Improving Well...

Thank you for your participation in the first round questionnaire of this study which aims to develop guidelines for optimising yoga interventions in reducing depression and anxiety. Participants have provided valuable information from their wealth of experience.

The information has been summarised, including areas of general agreement, as well as a range of differing points of view. Based on this summary, this second round questionnaire will attempt to bring further consensus to optimising intervention protocols.

IMPORTANT NOTE:
The development of these guidelines is NOT an attempt to develop a standardised treatment. Consistent with the traditional teachings of yoga, study participants have emphasised the importance of an individually tailored approach, that takes into consideration numerous individual factors including age, level of ability, severity of symptoms, general health, interest, motivation, and life circumstances.

By completing this round two questionnaire, you will further assist in the development of guidelines for suitably trained yoga teachers to design individually tailored yoga practices for people with depression or anxiety. Such practices would be designed to alleviate depression and anxiety, and increase positive emotions and well-being.

Completion of the questionnaire is again taken as acknowledgement of your consent to participate.

Thank you again for your participation.

EXPECTED BENEFITS

Questions 1 to 3 are about the EXPECTED BENEFITS of a suitable, individually tailored yoga practice, for people with depression or anxiety.

1. An individualised yoga practice is generally considered to be beneficial for people with mild or moderate depression or anxiety.

Yoga was not considered to be as beneficial for people with more severe depression or anxiety. However, some suggested that yoga may still be beneficial for people with severe depression or anxiety if it is individually tailored for each person, guided by an experienced teacher, and done in conjunction with other treatments.

Do you agree?

- Agree
- Disagree
- Not sure

Comments (optional)

EXPECTED BENEFITS

2. For people with MILD or MODERATE depression or anxiety, CONSIDERABLE benefit is generally expected from doing a suitable individually tailored yoga practice.

Benefits include reduction in symptoms and increases in positive emotion and well-being, such as
- improvements in sleep
- ability to wind down and relax
- more relaxed breathing
- greater sense of initiative
- greater sense of meaning and purpose
- increased frequency of positive emotions
- reduced irritability
- reduced physical symptoms, such as dryness of mouth, trembling of hands.

These improvements may also be measured using various scales for depression, anxiety, positive emotions and well-being.

How much benefit could be expected for a person with MILD or MODERATE depression or anxiety, if they do a suitable individually tailored yoga practice, under the guidance of an experienced teacher, and in conjunction with other forms of treatment?

	10%	20%	30%	40%	50%	60%	70%	80%	90%	100%
Expected benefit	○	○	○	○	○	○	○	○	○	○

Comments (optional)

3. For people with SEVERE depression or anxiety:

How much benefit could be expected for a person with SEVERE depression or anxiety, if they do a suitable individually tailored yoga practice, under the guidance of an experienced teacher, and in conjunction with other forms of treatment?

	10%	20%	30%	40%	50%	60%	70%	80%	90%	100%
Expected benefit	○	○	○	○	○	○	○	○	○	○

Comments (optional)

RECOMMENDED FREQUENCY AND DURATION OF YOGA PRACTICE

A number of recommendations have been made about HOW OFTEN and FOR HOW LONG a person would need to do the yoga, in order to gain benefit.

The yoga practice would be individually tailored in consultation with an experienced teacher, done regularly at home, and in conjunction with other treatments.

These are general recommendations only, and are summarised below. For each question 4 to 6, please indicate if you agree or disagree.

4. It is recommended for a person with depression or anxiety to do each session of their individually tailored yoga practice for....

	Agree	Disagree
AVERAGE of 30 to 40 minutes per session	○	○
MINIMUM of 15 minutes per session	○	○
MAXIMUM of 90 minutes per session	○	○

Comments (optional)

5. It is recommended for a person with depression or anxiety to do each session of their individually tailored yoga practice for....

	Agree	Disagree
AVERAGE of 5 to 6 sessions per week	○	○
MINIMUM of 4 sessions per week	○	○
MAXIMUM of 14 sessions per week (up to twice a day)	○	○

Comments (optional)

6. It is recommended for a person with depression or anxiety to do their yoga practice for at least 6 weeks.

○ Agree ○ Disagree

Comments (optional)

REDUCING DEPRESSION AND IMPROVING WELL-BEING

There are many components of yoga practice, that include various approaches and techniques. It is generally agreed that yoga is most beneficial when these different components or techniques are used with an integrated approach. That is, in combination or conjunction with each other. This integrated approach includes particular recommendations for each of the components and techniques being used.

It is recommended that various approaches and techniques are always done in accordance with the level of ability of each individual, and may also be beneficial in conjunction with other forms of treatment.

Questions 7 to 14 include the components of yoga practice that were recommended as beneficial for reducing depression, and improving well-being.

Whilst these tools or techniques are often used in combination or conjunction with other techniques, questions 7 to 10 focus in the basic approach for each of the main components themselves, which does not exclude the possibility of using different or additional components in combination.

7. The following approaches to YOGA POSTURES (asana) have been recommended as important components of yoga practice for reducing depression. This question seeks to clarify the relative importance of these factors.

For REDUCING DEPRESSION, how important is it that YOGA POSTURES are done with a focus on:

	Not Important	Somewhat Important	Very Important	Essential
Moving repetition of postures (rather than long holding)	◯	◯	◯	◯
Dynamic sequences of postures	◯	◯	◯	◯
A range range of different postures	◯	◯	◯	◯
Backward-bending postures	◯	◯	◯	◯
Chest and heart opening	◯	◯	◯	◯
Coordinated flow of breath with movement	◯	◯	◯	◯
Inhalation in postures and movement	◯	◯	◯	◯
Resting or relaxation postures	◯	◯	◯	◯
Appropriate level of challenge for ability of each individual	◯	◯	◯	◯

Comment (optional)

REDUCING DEPRESSION AND IMPROVING WELL-BEING

8. The following approaches to REGULATION OF BREATHING (pranayama) have been recommended as important components of yoga practice for reducing depression. This question seeks to clarify the relative importance of these factors.

For REDUCING DEPRESSION, how important is it that BREATH REGULATION is done with a focus on:

	Not Important	Somewhat Important	Very Important	Essential
Abdominal breathing	◯	◯	◯	◯
Inhalation	◯	◯	◯	◯
Comfortable retention after inhalation	◯	◯	◯	◯
Right nostril breathing	◯	◯	◯	◯
Right nostril breathing on inhalation	◯	◯	◯	◯
Rapid breathing techniques, such as kapalabhati	◯	◯	◯	◯
Cooling breath (sitali)	◯	◯	◯	◯
Always done according to individual abilities	◯	◯	◯	◯

Comment (optional)

REDUCING DEPRESSION AND IMPROVING WELL-BEING

9. The following approaches to RELAXATION have been recommended as important components of yoga practice for reducing depression. This question seeks to clarify the relative importance of these factors.

For REDUCING DEPRESSION, how important is it that RELAXATION is done with a focus on:

	Not Important	Somewhat Important	Very Important	Essential
Resting between and after postures	◯	◯	◯	◯
With legs elevated	◯	◯	◯	◯
Restorative (passive-supported) postures	◯	◯	◯	◯
Active focus on physical body (e.g. body-scan; progressive muscle relaxation), to shift focus away from mind and thoughts	◯	◯	◯	◯
Done with visualisations, that are positive expansive and energising, e.g. sun, open space,	◯	◯	◯	◯
Using guided relaxation techniques	◯	◯	◯	◯

Comment (optional)

REDUCING DEPRESSION AND IMPROVING WELL-BEING

10. The following approaches to MEDITATION have been recommended as important components of yoga practice for reducing depression. This question seeks to clarify the relative importance of these factors.

For REDUCING DEPRESSION, how important is it that MEDITATION is done with a focus on:

	Not Important	Somewhat Important	Very Important	Essential
Mindfulness (learning to focus attention on observing the present experience)	○	○	○	○
Active meditations, including moving, chanting, guided.	○	○	○	○
Something for the mind to do and focus on, rather than just observation (e.g. counting, repeated words or phrases (mantra); visualisation; image or symbol; candle gazing; smiling heart)	○	○	○	○
A concept, idea or value, such as something positive, energising, confidence building, gratitude.	○	○	○	○
Generally, not to be used for beginners, or only after person feels they have some ability to control their thoughts	○	○	○	○

Comment (optional)

REDUCING DEPRESSION AND IMPROVING WELL-BEING

11. All these components, and combinations of them, are considered to be beneficial for reducing depression.

The first questionnaire also asked participants to rate the IMPORTANCE of each component.

Using a rating scale from 1=not recommended to 5=essential, these components of yoga practice for reducing depression were rated by participants as either "very important" or "essential" as follows:

1st: Regulation of breathing (pranayama) (95% of participants, with an “importance” rating of 4.4)
2nd: Yoga postures (asana) (76% of participants, “importance” rating of 4.1)
3rd and 4th: Relaxation and meditation were rated similarly (55% and 57% of participants, with an “importance” rating of 3.8 and 3.6 respectively)

Do you agree with this overall rating of the relative importance of these components of yoga for reducing DEPRESSION?

- ○ YES, agree
- ○ NO, disagree

If "no", please rank them in order of relative importance

12. There was general consensus that this order of relative importance would be the same for using yoga to increase positive emotion and well-being in people with depression.

Do you agree?

- ○ YES, agree
- ○ NO, disgree

Comments (optional)

REDUCING DEPRESSION AND IMPROVING WELL-BEING

13. A variety of ADDITIONAL COMPONENTS or techniques of yoga practice are also recommended as important for reducing depression.

For REDUCING DEPRESSION, how important are these OTHER YOGA PRACTICES?

	Not Important	Somewhat Important	Very Important	Essential
Repetition of meaningful words or phrases (mantra)	○	○	○	○
Formulation of meaningful affirmations and intentions (samkalpa)	○	○	○	○
Visualisation and symbolic imagery techniques (bhavana)	○	○	○	○
Cultivation of positive values, attitudes and behaviours (including gratitude, kindness, compassion, forgiveness)	○	○	○	○
Sound or chanting (from any suitable language or culture)	○	○	○	○
Symbolic gesture	○	○	○	○
Awareness of negative sensory input (including TV, movies, music, literature, multi-media, news)	○	○	○	○
Spirituality and prayer	○	○	○	○

Comment (optional)

REDUCING DEPRESSION AND IMPROVING WELL-BEING

14. A number of OTHER FACTORS related to “YOGIC VALUES AND LIFESTYLE” are also recommended for reducing depression.

For REDUCING DEPRESSION, how important are these OTHER FACTORS OF YOGIC LIFESTYLE?

	Not Important	Somewhat Important	Very Important	Essential
Lifestyle factors, including diet, smoking, drugs and alcohol, sleep, work, exercise	○	○	○	○
Social involvement and support - linking with a supportive community	○	○	○	○
Positive relationships	○	○	○	○
Group yoga classes	○	○	○	○
Education about yoga teachings, and the potential benefits of yoga	○	○	○	○
Developing self-empowering knowledge	○	○	○	○
Service to others, including volunteer work	○	○	○	○
Pleasing environment, free from clutter, with good ventilation and natural light	○	○	○	○
Exposure to sunlight and natural environments	○	○	○	○
Regular laughing	○	○	○	○

Comment (optional)

REDUCING DEPRESSION AND IMPROVING WELL-BEING

15. For people with depression, a number of factors were identified as important to AVOID.

How important is it to AVOID the following yoga practices and techniques for people with depression?

	OK to include	Important to Avoid	Very Important to Avoid	Essential to Avoid
Longer holds in forward-bending postures	◯	◯	◯	◯
Any strong or strenuous postures, if there is low motivation and energy	◯	◯	◯	◯
Heated or strenuous styles of yoga	◯	◯	◯	◯
Sedentary, resting or relaxing postures	◯	◯	◯	◯
Breath regulation that focuses on long exhales and holding after exhales	◯	◯	◯	◯
Rapid breathing techniques, such as kapalabhati, if anxiety or history of trauma is also present	◯	◯	◯	◯
Breathing techniques with left nostril dominance	◯	◯	◯	◯
Long relaxation practices	◯	◯	◯	◯
Meditation practices with eyes closed	◯	◯	◯	◯
Meditation practices that are internalising	◯	◯	◯	◯
Meditation practices that are unguided	◯	◯	◯	◯
Meditation practices without any specific focus (such as emptiness or inner silence meditation)	◯	◯	◯	◯
Any practices which are too introspective	◯	◯	◯	◯
Standardised approaches (prescriptive methods)	◯	◯	◯	◯

Comment (optional)

REDUCING ANXIETY AND IMPROVING WELL-BEING

The previous section was for the reduction of depression and improving well-being. The following section is for the reduction of anxiety and improving well-being. There are many similarities in approach, and some important differences.

Again, it is emphasised that yoga is most beneficial when the different components or techniques are used with an integrated approach. That is, in combination or conjunction with each other. This integrated approach also includes particular recommendations for each of the components and techniques being used.

It is recommended that various approaches and techniques are always done in accordance with the level of ability of each individual, and may also be beneficial in conjunction with other forms of treatment.

Questions 16 to 23 include the components of yoga practice that were recommended as beneficial for reducing ANXIETY, and improving well-being.

Whilst these tools or techniques are often used in combination or conjunction with other techniques,
questions 16 to 19 focus in the basic approach for each of the main components themselves, which does not exclude the
possibility of using different or additional components in combination.

16. The following approaches to YOGA POSTURES (asana) have been recommended as important components of yoga practice for reducing anxiety. This question seeks to clarify the relative importance of these factors.

For REDUCING ANXIETY, how important is it that YOGA POSTURES are done with a focus on:

	Not Important	Somewhat Important	Very Important	Essential
Simple, gentle sequences of postures	◯	◯	◯	◯
More dynamic to begin, gradually slowing down	◯	◯	◯	◯
Relaxed and restorative stay positions, seated or lying down	◯	◯	◯	◯
A calming effect	◯	◯	◯	◯
Coordinated flow of breath with movement	◯	◯	◯	◯
Relaxing chest, abdomen and diaphragm areas	◯	◯	◯	◯
Forward-bending postures	◯	◯	◯	◯
Variety of postures, including back bending, twisting, standing balance, inversions	◯	◯	◯	◯
Exhalation in postures and movement (more on breath regulation in Q15)	◯	◯	◯	◯
Resting or relaxation postures (more on relaxation in Q16)	◯	◯	◯	◯
Appropriate level of challenge for ability of each individual	◯	◯	◯	◯

Comment (optional)

REDUCING ANXIETY AND IMPROVING WELL-BEING

17. The following approaches to REGULATION OF BREATHING (pranayama) have been recommended as an important component of yoga practice for reducing anxiety. This question seeks to clarify the relative importance of these factors.

For REDUCING ANXIETY, how important is it that BREATH REGULATION is done with a focus on:

	Not Important	Somewhat Important	Very Important	Essential
Abdominal breathing, in a relaxed lying down or seated position	○	○	○	○
Regulating the breath to become steady	○	○	○	○
Lengthening exhalation	○	○	○	○
Comfortable holding after exhalation	○	○	○	○
Left nostril breathing	○	○	○	○
Left nostril breathing on exhalation	○	○	○	○
Alternate nostril breathing	○	○	○	○
Cooling breath (sitali)	○	○	○	○
“humming bee” breath (brahmari)	○	○	○	○

Comment (optional)

REDUCING ANXIETY AND IMPROVING WELL-BEING

18. The following approaches to to RELAXATION have been recommended as an important component of yoga practice for reducing anxiety. This question seeks to clarify the relative importance of these factors.

For REDUCING ANXIETY, how important is it that RELAXATION is done with a focus on:

	Not Important	Somewhat Important	Very Important	Essential
Resting between and after postures	○	○	○	○
In relaxed lying down position of stillness	○	○	○	○
With legs elevated	○	○	○	○
Restorative (passive-supported) postures	○	○	○	○
Abdominal breathing, lengthening exhale	○	○	○	○
Guided relaxation (e.g. of body parts, progressive muscle relaxation, body-awareness scan) to shift focus away from mind and thoughts	○	○	○	○
Being done with visualisations, that have a calming effect	○	○	○	○

Comment (optional)

REDUCING ANXIETY AND IMPROVING WELL-BEING

19. The following approaches to MEDITATION have been recommended as an important component of yoga practice for reducing anxiety. This question seeks to clarify the relative importance of these factors.

For REDUCING ANXIETY, how important is it that MEDITATION is done with a focus on:

	Not Important	Somewhat Important	Very Important	Essential
Mindfulness (learning to focus attention on observing the present experience)	○	○	○	○
Active meditations, including moving, chanting, guided.	○	○	○	○
Something for the mind to do, rather than just observation (e.g. counting, repeated words or phrases (mantra); visualisation; image or symbol; candle gazing; smiling heart)	○	○	○	○
A concept, idea or value, such as something positive, calming, confidence building, gratitude.	○	○	○	○
Generally, not to be used for beginners, or only after person feels they have some ability to control their thoughts.	○	○	○	○

Comment (optional)

REDUCING ANXIETY AND IMPROVING WELL-BEING

20. All these components, and combinations of them, are considered to be beneficial for reducing anxiety.

The first questionnaire also asked participants to rate the importance of each component.

Using a rating scale from 1=not recommended to 5=essential, these components of yoga practice for reducing anxiety were rated by participants as either "very important" or "essential" as follows:

1st: Relaxation (95% of participants, with an "importance" rating of 4.5)
2nd: Regulation of Breathing (pranayama) (89% of participants, "importance" rating of 4.3)
3rd: Meditation (84% of participants, "importance" rating of 4.1)
4th: Postures (asana) (63% of participants, "importance" rating of 3.9)

Do you agree with this overall rating of the relative importance of these components of yoga for reducing ANXIETY?

- YES, agree
- NO, disagree

If "no", please rank them in order of importance

21. There was general consensus that this order of relative importance would be the same for using yoga to increase positive emotion and well-being in people with anxiety. Do you agree?

- YES, agree
- NO, disagree

Comments (optional)

REDUCING ANXIETY AND IMPROVING WELL-BEING

22. A variety of additional components or techniques of yoga practice are also recommended as important for reducing anxiety.

For REDUCING ANXIETY, how important are these OTHER YOGA PRACTICES?

	Not Important	Somewhat Important	Very Important	Essential
Repetition of meaningful words or phrases (mantra)	○	○	○	○
Formulation of meaningful affirmations and intentions (samkalpa)	○	○	○	○
Visualisation and symbolic imagery techniques (bhavana), for a calming and focusing effect	○	○	○	○
Cultivation of positive values, attitudes and behaviours (including gratitude, kindness, compassion, forgiveness)	○	○	○	○
Sound or chanting (from any suitable language or culture)	○	○	○	○
Symbolic gesture	○	○	○	○
Awareness of negative sensory input (including TV, movies, music, literature, multi-media, news)	○	○	○	○
Spirituality and prayer	○	○	○	○
Other	○	○	○	○

Comment (optional)

REDUCING ANXIETY AND IMPROVING WELL-BEING

23. A number of OTHER FACTORS related to “YOGIC VALUES AND LIFESTYLE” are also recommended for reducing anxiety.

For REDUCING ANXIETY, how important are these OTHER FACTORS OF YOGIC LIFESTYLE?

	Not Important	Somewhat Important	Very Important	Essential
Lifestyle factors, including diet, caffeine, smoking, drugs and alcohol, sleep, work, self-care.	○	○	○	○
Social involvement and support; linking with a supportive community;	○	○	○	○
Positive relationships	○	○	○	○
Education about yoga teachings, and the potential benefits of yoga	○	○	○	○
Developing self-empowering knowledge	○	○	○	○
Mindfulness in daily life	○	○	○	○
Service to others, including volunteer work	○	○	○	○
Pleasing environment, free from clutter, with good ventilation and natural light	○	○	○	○
Exposure to sunlight and natural environment	○	○	○	○

Comment (optional)

REDUCING ANXIETY AND IMPROVING WELL-BEING

24. For people with anxiety, a number of factors were identified as important to AVOID.

How important is it to AVOID the following yoga practices and techniques for people with anxiety?

	OK to include	Important to Avoid	Very Important to Avoid	Essential to Avoid
Breath regulation that focuses on inhalation	◯	◯	◯	◯
Breath regulation that focuses on holding after inhalation	◯	◯	◯	◯
Breath regulation that focuses on holding after exhalation	◯	◯	◯	◯
Rapid breathing techniques, such as kapalabhati or bhastrika	◯	◯	◯	◯
Breathing techniques with right nostril dominance	◯	◯	◯	◯
Any focus on the breath, especially in sitting practices	◯	◯	◯	◯
Focus on backward bending postures	◯	◯	◯	◯
Techniques that require difficult and complex instructions	◯	◯	◯	◯
Vigorous or strenuous postures and sequences, that are over-stimulating or arousing	◯	◯	◯	◯
Techniques that emphasise ability, accomplishment, performance, competition	◯	◯	◯	◯
Yoga done in heated, crowded or enclosed spaces	◯	◯	◯	◯
Techniques done with eyes closed	◯	◯	◯	◯
Meditation practices that are unguided	◯	◯	◯	◯
Meditation practices without any specific focus (such as emptiness or inner silence meditation)	◯	◯	◯	◯
Standardised approaches (prescriptive methods)	◯	◯	◯	◯

Comment (optional)

TRAINING AND EXPERIENCE

When teaching yoga to people with mental health concerns, such as depression or anxiety, it is considered essential that yoga teachers have suitable training and experience.

Training and experience in other related professions, such as psychology or psychiatry, and complementary health, would be considered valuable, but not essential, for teaching yoga in the area of mental health.

The following are recommendations for minimum yoga training and teaching experience.

Please indicate whether or not you agree with these recommendations.

25. A minimum of 500 hours of yoga teacher training, over a minimum of 2 years.

- YES, agree
- NO, disagree

Comment (optional)

26. A minimum of 2 years teaching experience

- YES, agree
- NO, disagree

Comment (optional)

27. Specific training in developing individually tailored yoga practices (rather than general training for teaching group classes), was rated by 95% of participants as either "very important" or "essential".

Do you agree with this recommendation?

- YES, agree
- NO, disagree

Comment (optional)

28. Specific training in yoga for mental health (rated "very important" or "essential" by 89% of study participants).

Do you agree with this recommendation?

- YES, agree
- NO, disagree

Comment (optional)

29. Professional supervision or mentoring for yoga teachers who are working in the area of mental health (rated very important or essential by 89% of study participants).

Do you agree with this recommendation?

- YES, agree
- NO, disagree

Comment (optional)

30. Any further comments?

Thank you for your participation. Your contribution is greatly appreciated.

We will contact you again soon, for the third round of the study.
This will be a brief and final series of questions to confirm any of areas requiring further consensus.
